# The Prince and the Hospitals

**Published:** 1902-03-15

**Authors:** 


### The Hospital

A Journal of

The Medical Sciences and Hospital Administration.

Vol. XXXI.—No. 807.] Edited by Sir Henry Burdett, K.C.B., and Solomon C. Smith, M.D., M.R.C.P. [March 15, 1902.

### The Prince and the Hospitals.

The dawn of the twentieth century has shown in a marked degree the effects of example upon many classes of people who have been associated with hospitals in various capacities. For many years a few zealous men devoted a very large amount of time and personal service to the cause of certain hospitals, and, in a few instances, to the whole hospital question. This active interest on the part of the few, and its recognition, has stimulated effort on the part of others, and to day there are signs that within the next decade the office of president of a hospital will cease to be a sinecure, and will be regarded by each holder as demanding a measure of active personal service. The influence wielded by the Prince of Wales is of course great, and it is matter for congratulation that with the Princess of Wales he should have determined to periodically inspect the hospitals of which the Prince is president. Last week Their Royal Highnesses paid an unexpected visit to the Great Northern Central Hospital, parts of which they thoroughly inspected, and at the conclusion of their visit they intimated that they should return to the hospital again on a future day in order to view those portions of the buildings which they had not yet seen. The example of the Prince will, we expect, be largely followed by the other presidents of hospitals, and we hope before long to find that hospital governors will determine not to re-elect to the office of president anyone who does not devote a measure of personal service to the hospital by making periodical inspections during his term of office.

This new departure made by the Prince of Wales is especially noteworthy at this time from the circumstance that the public duties devolving upon him are unusually heavy. Indeed, the members of the Royal Family at present available for public duty are much fewer than at any period during recent years. The Duke of Cambridge, though willing, is too old to be able to undertake active work of this description ; the Duke of Connaught is in Ireland, and so the Prince of Wales, for the present at any rate, is almost single-handed in the matter. Trained in the Navy, with a keen sense of duty, an active temperament, a mastery of details, and a thoroughness in work equal to that of the late Lord Randolph Churchill, the Prince of Wales is entitled to be regarded as one of the most active forces of the day. He shares his father's whole-hearted interest in hospitals and kindred institutions, and his determination to make the office of president an active power for good is as noteworthy as it is full of hope for the future.

In intimating to the Council of King Edward's Fund that he looked upon it as a great honour that the King should have selected him as President of that Fund, the Prince of Wales stated that it would be his earnest endeavour to complete the work which his father had initiated. With this object in view he had devoted a good deal of time to the consideration of how the permanent income of the Fund could be increased say, by, £50,000 a year. In the result the Prince determined to himself attempt to interest a large number of wealthy and influential people who have heretofore taken little or no part in the work of the Fund. Within a couple of months he has succeeded in procuring promises of support, which in the aggregate have produced a sum which will yield from investments £20,000 a year. The Prince went on to declare that it would be a matter of supreme satisfaction to himself if during his first year of office the income could be increased to at least £100,000. That is the task voluntarily undertaken, in addition to his many and pressing public duties, by the Prince of Wales.

That H.R.H. should have succeeded in procuring by his own efforts a permanent revenue of £20,000 a year for the London hospitals in the space of two months is a remarkable fact, unprecedented we believe in the work of any single man in the field of voluntary charity. Those who have had most to do with the raising of money for hospitals will at once appreciate the amount of time, thought, influence and devotion on the part of the Prince which this additional £20,000 a year represents. We feel confident that everybody will be stimulated by the Prince's example to use such influence as they possess in their own circle to secure promises of annual subscriptions, or donations of substantial amount for the investment fund, in order that they may be sent direct to the Prince of Wales in practical testimony of the warm appreciation which his spontaneous efforts have aroused. How unique is the service already rendered by the Prince of Wales will be apparent from the statement that the combined efforts of all the influential and active workers who co-operated to help the King's Fund in the first year of its establishment, only succeeded in raising a revenue of about £26,000 a year. That one man in two months' time should quietly, and by his own efforts, have raised £20,000 a year of revenue, as the Prince of Wales has done, is a fact, which should for ever endear him to the hospitals, and secure him the confidence and gratitude of his countrymen throughout the Empire.

